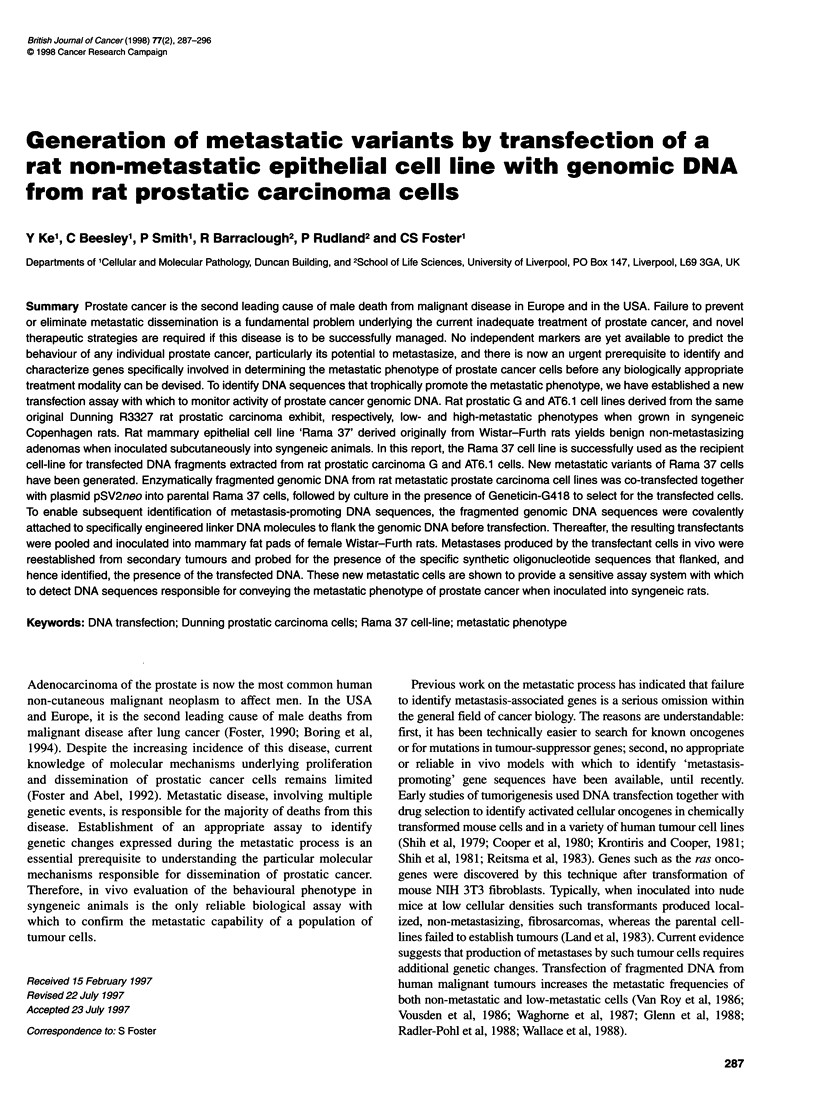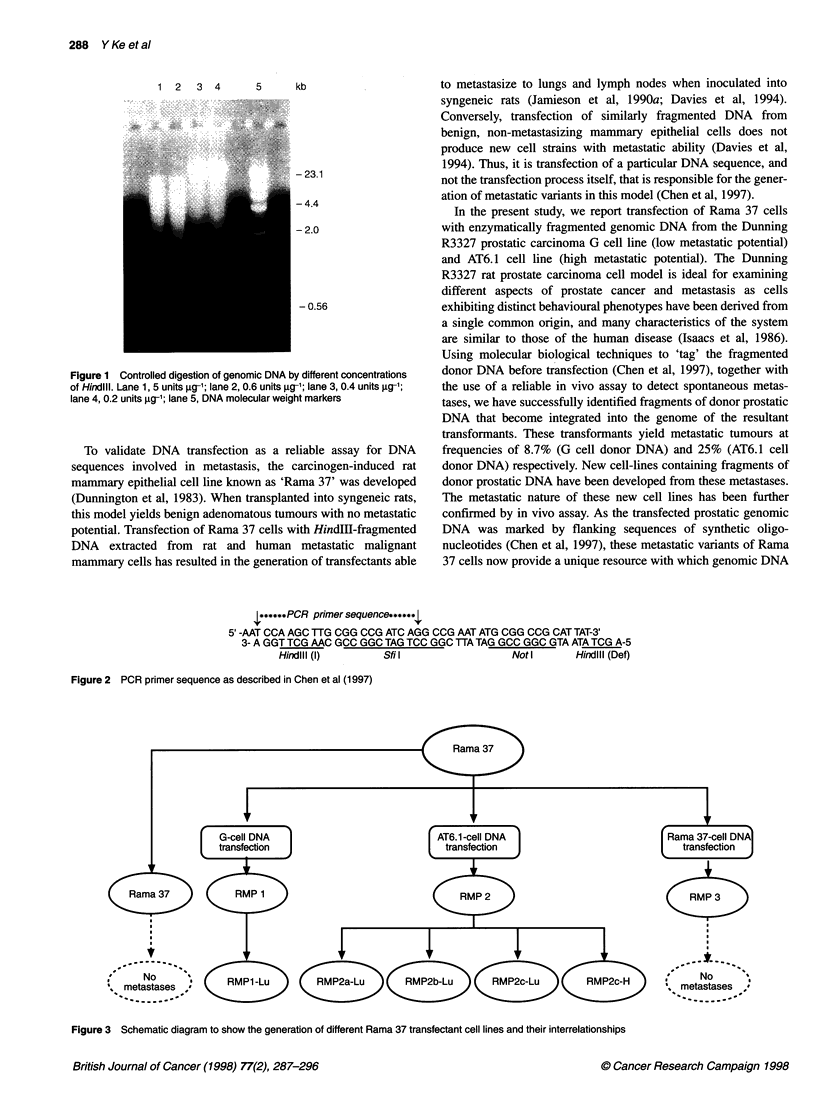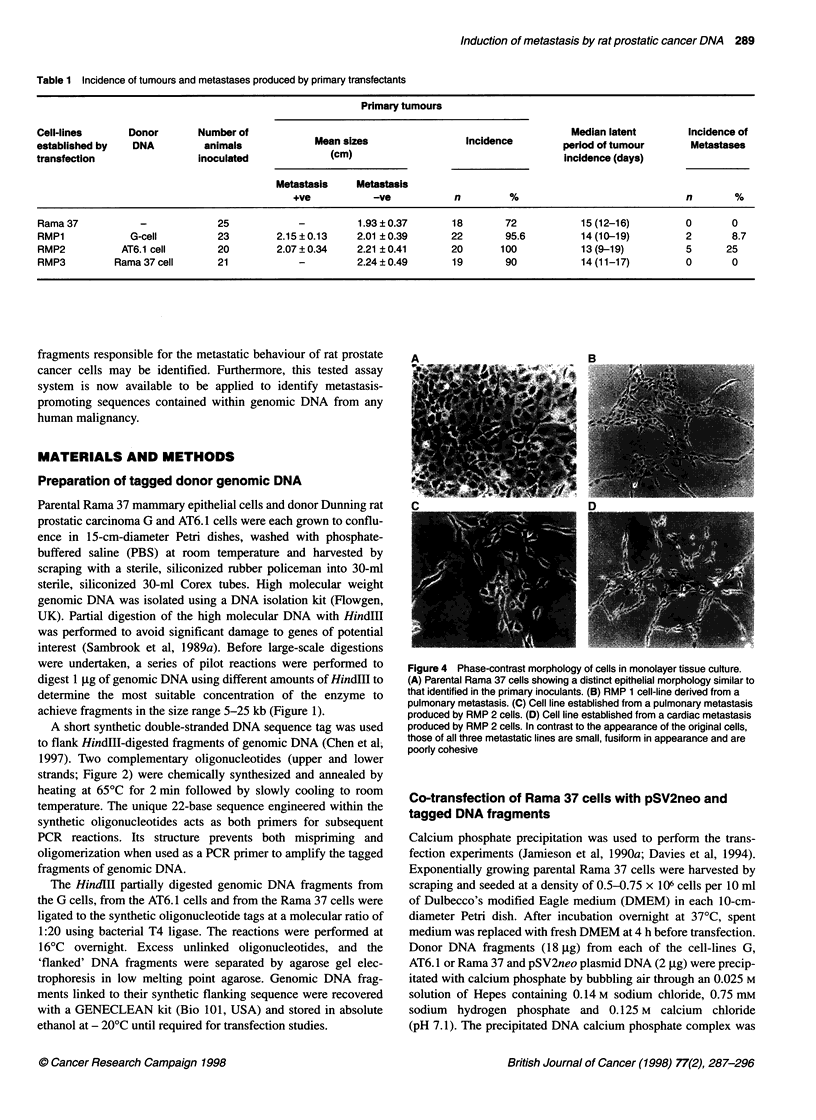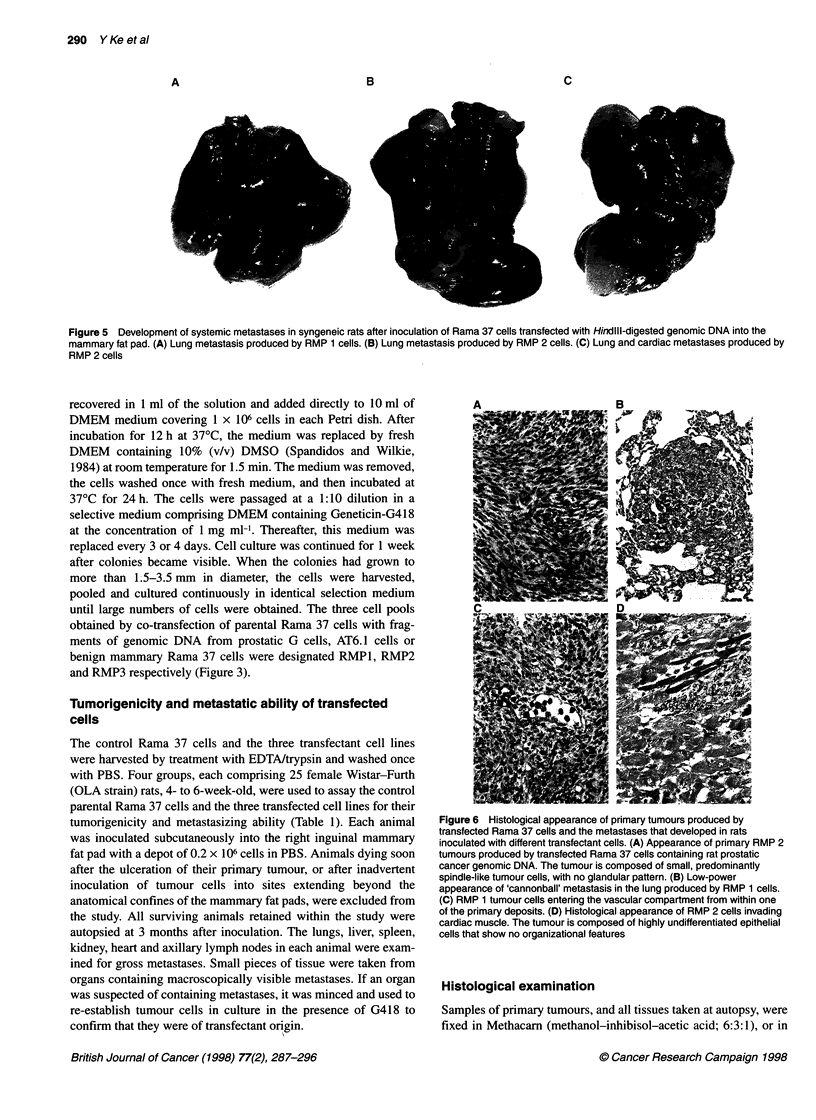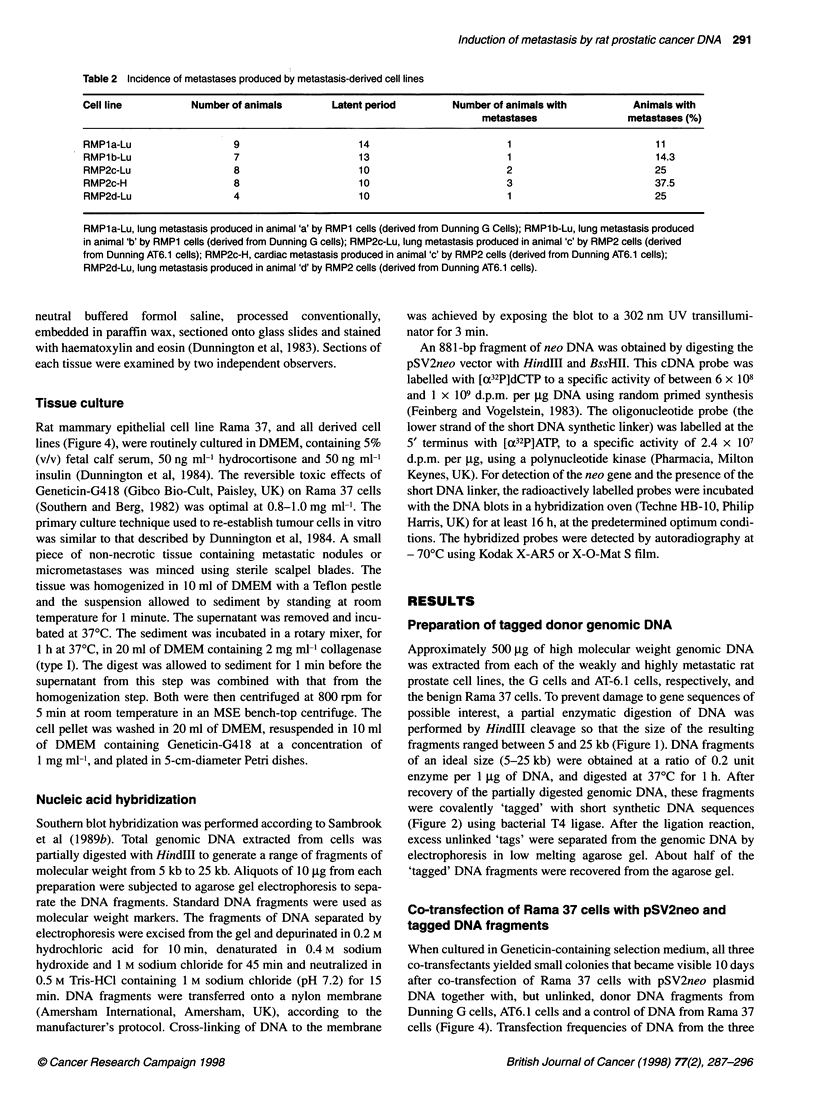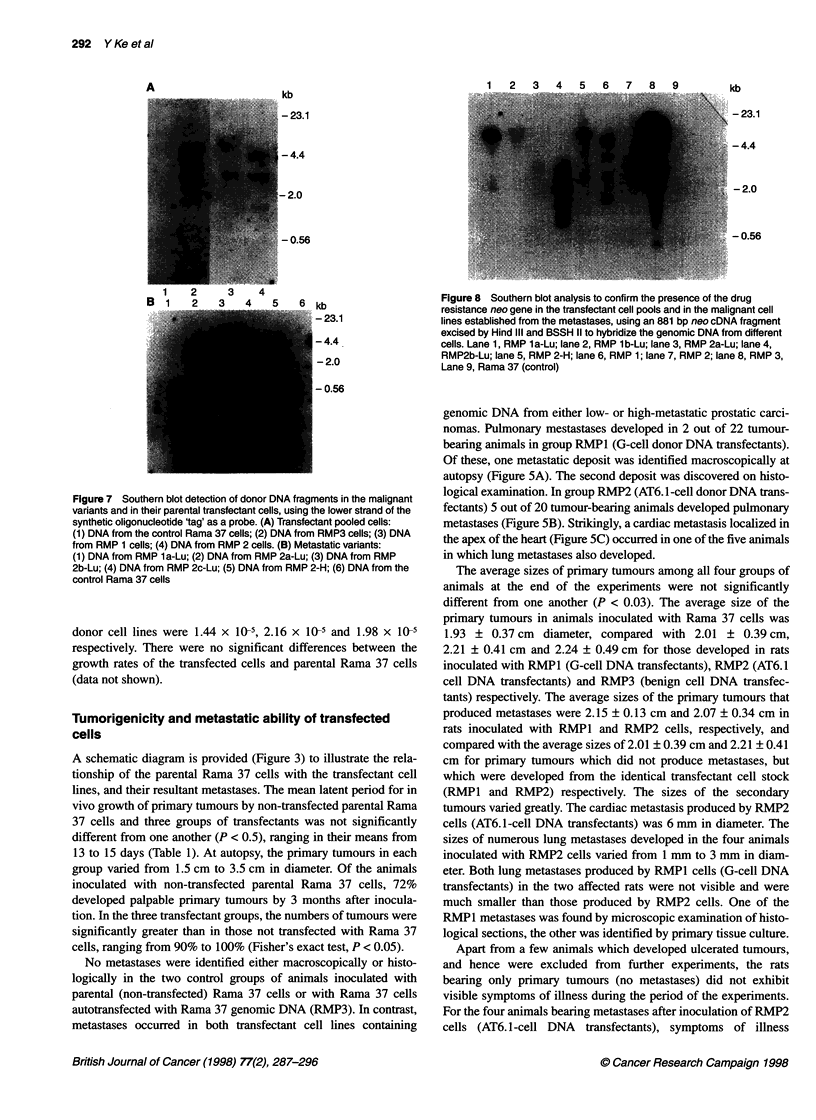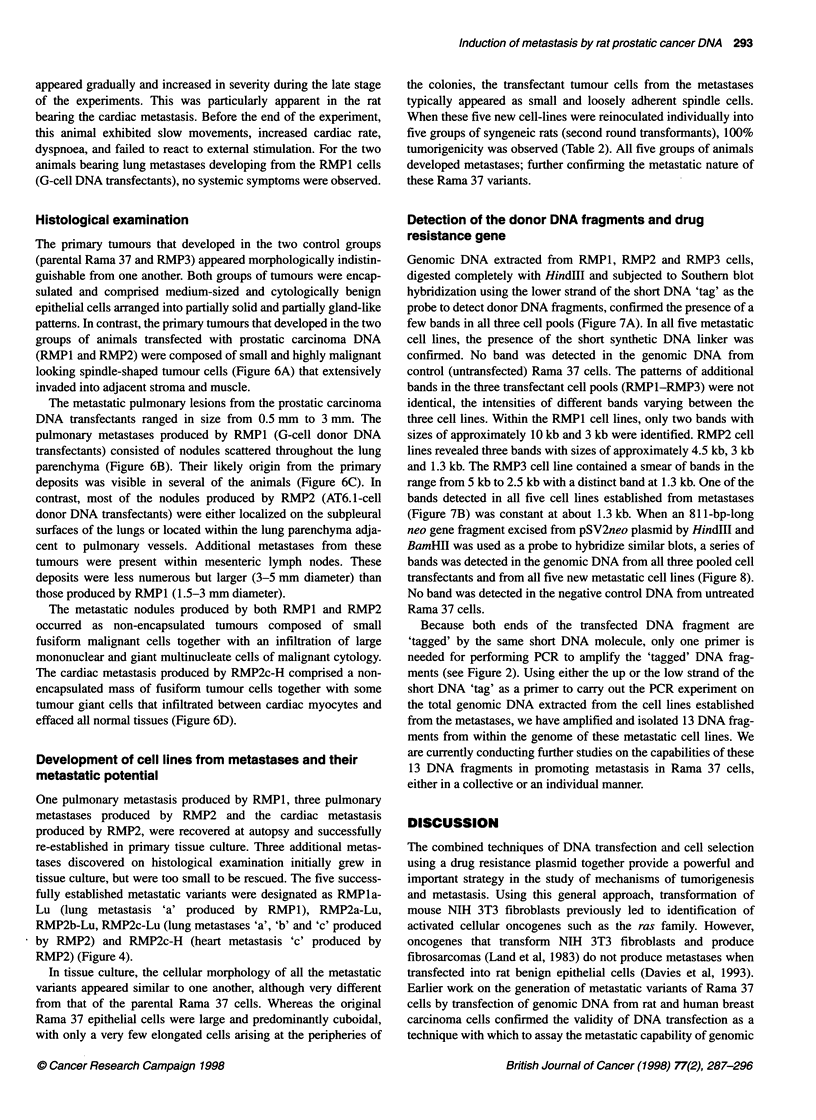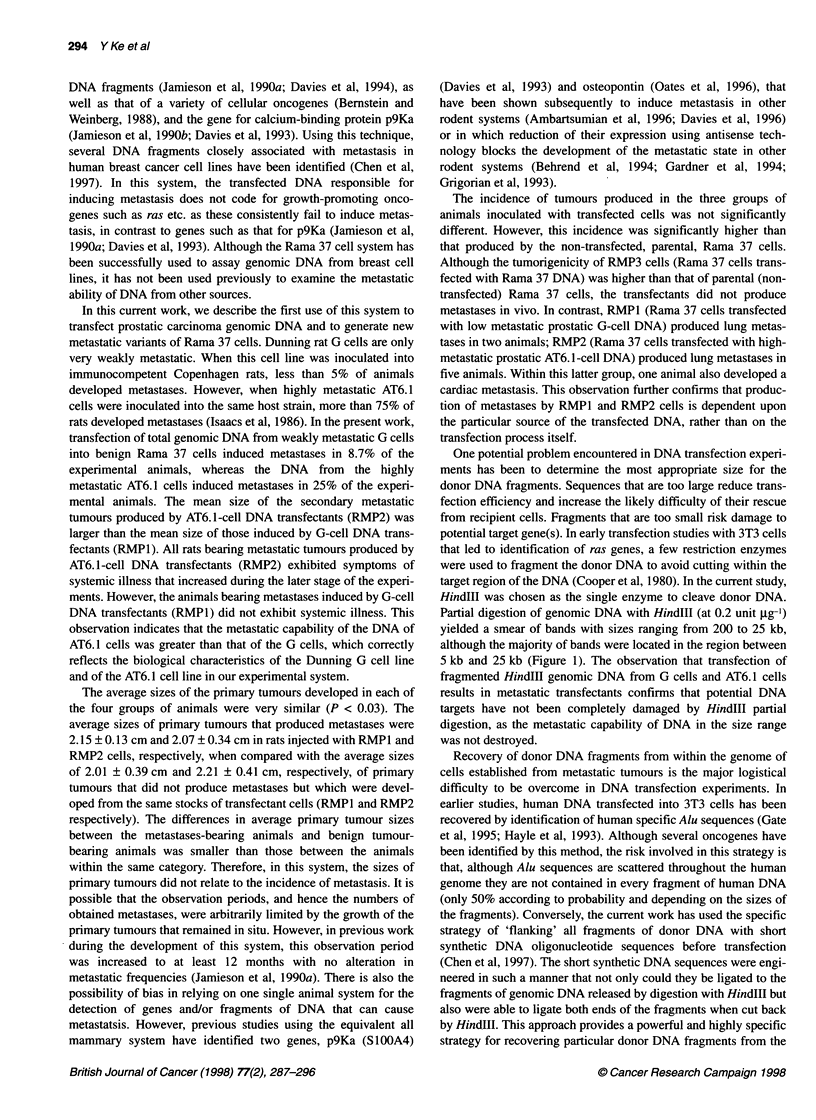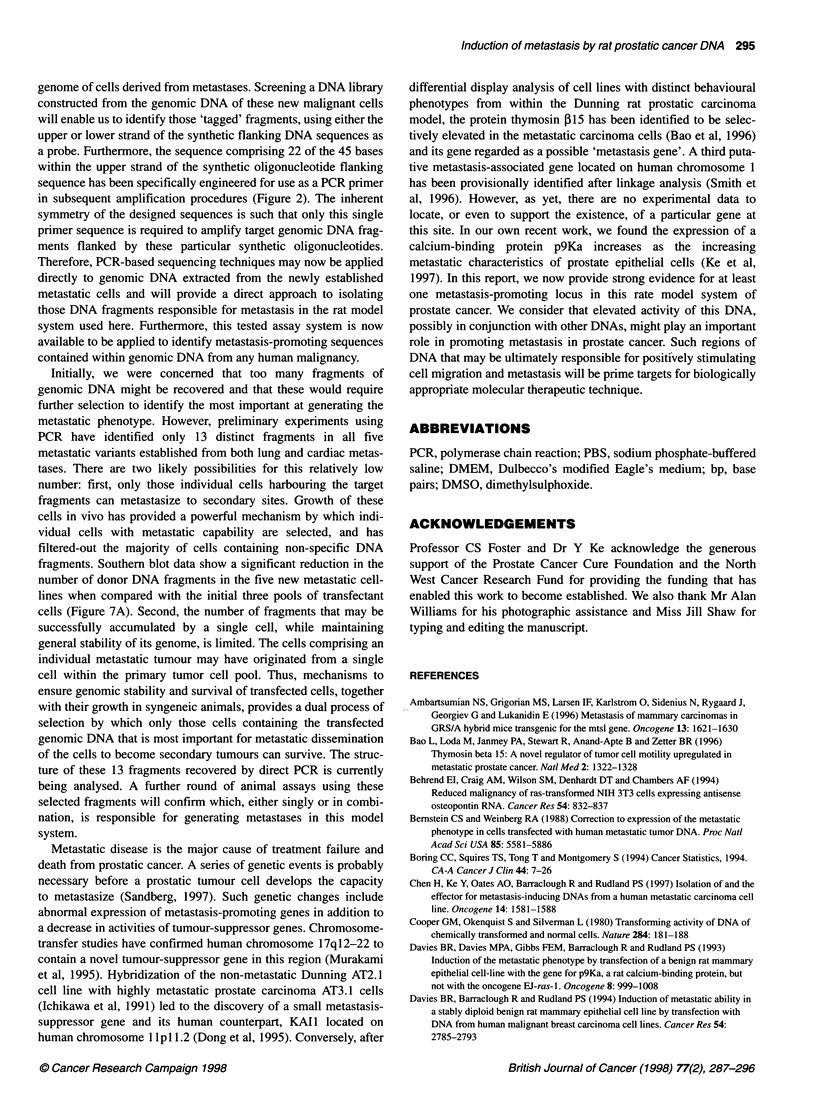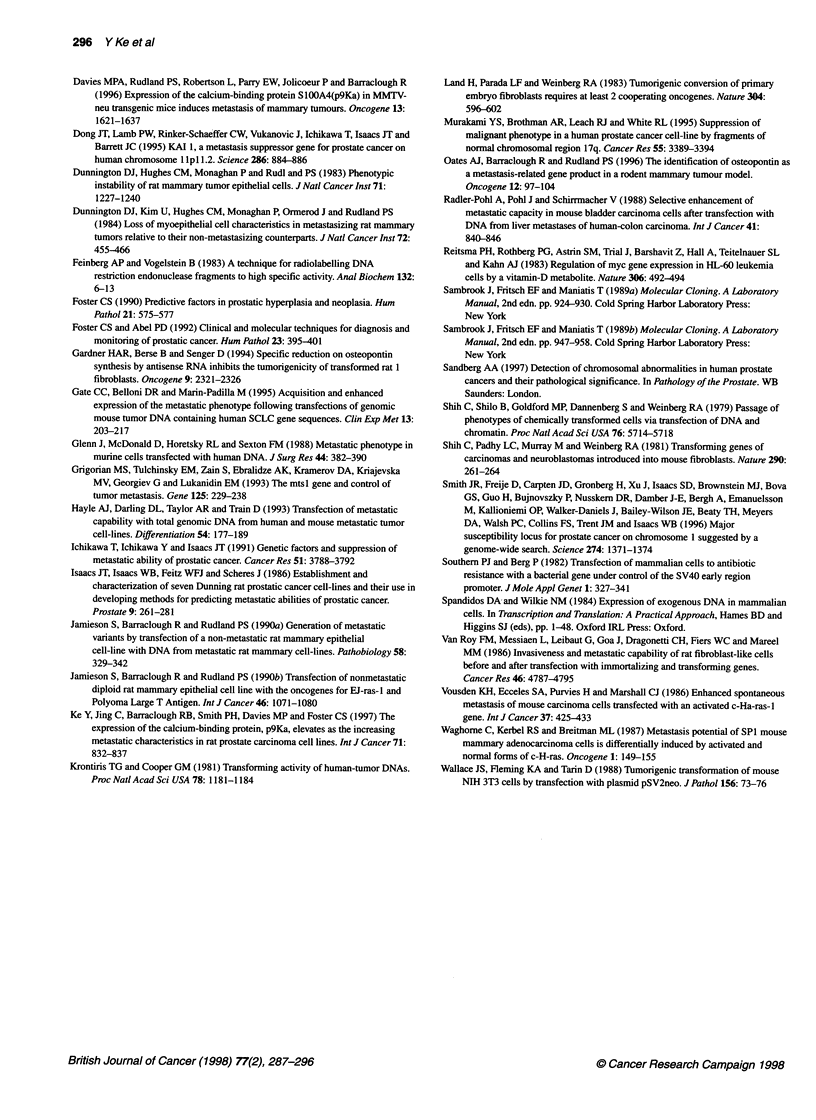# Generation of metastatic variants by transfection of a rat non-metastatic epithelial cell line with genomic DNA from rat prostatic carcinoma cells.

**DOI:** 10.1038/bjc.1998.45

**Published:** 1998

**Authors:** Y. Ke, C. Beesley, P. Smith, R. Barraclough, P. Rudland, C. S. Foster

**Affiliations:** Department of Cellular and Molecular Pathology, University of Liverpool, UK.

## Abstract

**Images:**


*British Journal of Cancer* (1998) **77**(2), 287–296
© 1998 Cancer Research Campaign

### Generation of metastatic variants by transfection of a rat non-metastatic epithelial cell line with genomic DNA from rat prostatic carcinoma cells

**Y Ke[1], C Beesley[1], P Smith[1], R Barraclough[2], P Rudland[2] and CS Foster[1]**

Departments of [1]Cellular and Molecular Pathology, Duncan Building, and [2]School of Life Sciences, University of Liverpool, PO Box 147, Liverpool, L69 3GA, UK

**Summary** Prostate cancer is the second leading cause of male death from malignant disease in Europe and in the USA. Failure to prevent or eliminate metastatic dissemination is a fundamental problem underlying the current inadequate treatment of prostate cancer, and novel therapeutic strategies are required if this disease is to be successfully managed. No independent markers are yet available to predict the behaviour of any individual prostate cancer, particularly its potential to metastasize, and there is now an urgent prerequisite to identify and characterize genes specifically involved in determining the metastatic phenotype of prostate cancer cells before any biologically appropriate treatment modality can be devised. To identify DNA sequences that trophically promote the metastatic phenotype, we have established a new transfection assay with which to monitor activity of prostate cancer genomic DNA. Rat prostatic G and AT6.1 cell lines derived from the same original Dunning R3327 rat prostatic carcinoma exhibit, respectively, low- and high-metastatic phenotypes when grown in syngeneic Copenhagen rats. Rat mammary epithelial cell line 'Rama 37' derived originally from Wistar–Furth rats yields benign non-metastasizing adenomas when inoculated subcutaneously into syngeneic animals. In this report, the Rama 37 cell line is successfully used as the recipient cell-line for transfected DNA fragments extracted from rat prostatic carcinoma G and AT6.1 cells. New metastatic variants of Rama 37 cells have been generated. Enzymatically fragmented genomic DNA from rat metastatic prostate carcinoma cell lines was co-transfected together with plasmid pSV2*neo* into parental Rama 37 cells, followed by culture in the presence of Geneticin-G418 to select for the transfected cells. To enable subsequent identification of metastasis-promoting DNA sequences, the fragmented genomic DNA sequences were covalently attached to specifically engineered linker DNA molecules to flank the genomic DNA before transfection. Thereafter, the resulting transfectants were pooled and inoculated into mammary fat pads of female Wistar–Furth rats. Metastases produced by the transfectant cells in vivo were reestablished from secondary tumours and probed for the presence of the specific synthetic oligonucleotide sequences that flanked, and hence identified, the presence of the transfected DNA. These new metastatic cells are shown to provide a sensitive assay system with which to detect DNA sequences responsible for conveying the metastatic phenotype of prostate cancer when inoculated into syngeneic rats.

**Keywords:** DNA transfection; Dunning prostatic carcinoma cells; Rama 37 cell-line; metastatic phenotype

Adenocarcinoma of the prostate is now the most common human non-cutaneous malignant neoplasm to affect men. In the USA and Europe, it is the second leading cause of male deaths from malignant disease after lung cancer (Foster, 1990; Boring et al, 1994). Despite the increasing incidence of this disease, current knowledge of molecular mechanisms underlying proliferation and dissemination of prostatic cancer cells remains limited (Foster and Abel, 1992). Metastatic disease, involving multiple genetic events, is responsible for the majority of deaths from this disease. Establishment of an appropriate assay to identify genetic changes expressed during the metastatic process is an essential prerequisite to understanding the particular molecular mechanisms responsible for dissemination of prostatic cancer. Therefore, in vivo evaluation of the behavioural phenotype in syngeneic animals is the only reliable biological assay with which to confirm the metastatic capability of a population of tumour cells.

*Received 15 February 1997*
*Revised 22 July 1997*
*Accepted 23 July 1997*

*Correspondence to:* S Foster

Previous work on the metastatic process has indicated that failure to identify metastasis-associated genes is a serious omission within the general field of cancer biology. The reasons are understandable: first, it has been technically easier to search for known oncogenes or for mutations in tumour-suppressor genes; second, no appropriate or reliable in vivo models with which to identify 'metastasis-promoting' gene sequences have been available, until recently. Early studies of tumorigenesis used DNA transfection together with drug selection to identify activated cellular oncogenes in chemically transformed mouse cells and in a variety of human tumour cell lines (Shih et al, 1979; Cooper et al, 1980; Krontiris and Cooper, 1981; Shih et al, 1981; Reitsma et al, 1983). Genes such as the *ras* oncogenes were discovered by this technique after transformation of mouse NIH 3T3 fibroblasts. Typically, when inoculated into nude mice at low cellular densities such transformants produced localized, non-metastasizing, fibrosarcomas, whereas the parental cell-lines failed to establish tumours (Land et al, 1983). Current evidence suggests that production of metastases by such tumour cells requires additional genetic changes. Transfection of fragmented DNA from human malignant tumours increases the metastatic frequencies of both non-metastatic and low-metastatic cells (Van Roy et al, 1986; Vousden et al, 1986; Waghorne et al, 1987; Glenn et al, 1988; Radler-Pohl et al, 1988; Wallace et al, 1988).

**Figure 1** Controlled digestion of genomic DNA by different concentrations of *Hin*dIII. Lane 1, 5 units μg$^{-1}$; lane 2, 0.6 units μg$^{-1}$; lane 3, 0.4 units μg$^{-1}$; lane 4, 0.2 units μg$^{-1}$; lane 5, DNA molecular weight markers

To validate DNA transfection as a reliable assay for DNA sequences involved in metastasis, the carcinogen-induced rat mammary epithelial cell line known as ‘Rama 37’ was developed (Dunnington et al, 1983). When transplanted into syngeneic rats, this model yields benign adenomatous tumours with no metastatic potential. Transfection of Rama 37 cells with *Hin*dIII-fragmented DNA extracted from rat and human metastatic malignant mammary cells has resulted in the generation of transfectants able to metastasize to lungs and lymph nodes when inoculated into syngeneic rats (Jamieson et al, 1990*a*; Davies et al, 1994). Conversely, transfection of similarly fragmented DNA from benign, non-metastasizing mammary epithelial cells does not produce new cell strains with metastatic ability (Davies et al, 1994). Thus, it is transfection of a particular DNA sequence, and not the transfection process itself, that is responsible for the generation of metastatic variants in this model (Chen et al, 1997).

In the present study, we report transfection of Rama 37 cells with enzymatically fragmented genomic DNA from the Dunning R3327 prostatic carcinoma G cell line (low metastatic potential) and AT6.1 cell line (high metastatic potential). The Dunning R3327 rat prostate carcinoma cell model is ideal for examining different aspects of prostate cancer and metastasis as cells exhibiting distinct behavioural phenotypes have been derived from a single common origin, and many characteristics of the system are similar to those of the human disease (Isaacs et al, 1986). Using molecular biological techniques to ‘tag’ the fragmented donor DNA before transfection (Chen et al, 1997), together with the use of a reliable in vivo assay to detect spontaneous metastases, we have successfully identified fragments of donor prostatic DNA that become integrated into the genome of the resultant transformants. These transformants yield metastatic tumours at frequencies of 8.7% (G cell donor DNA) and 25% (AT6.1 cell donor DNA) respectively. New cell-lines containing fragments of donor prostatic DNA have been developed from these metastases. The metastatic nature of these new cell lines has been further confirmed by in vivo assay. As the transfected prostatic genomic DNA was marked by flanking sequences of synthetic oligonucleotides (Chen et al, 1997), these metastatic variants of Rama 37 cells now provide a unique resource with which genomic DNA

**Figure 2** PCR primer sequence as described in Chen et al (1997)

**Figure 3** Schematic diagram to show the generation of different Rama 37 transfectant cell lines and their interrelationships

© Cancer Research Campaign 1998

**Table 1** Incidence of tumours and metastases produced by primary transfectants

| Cell-lines established by transfection | Donor DNA | Number of animals inoculated | Primary tumours: Mean sizes (cm) | | Incidence | | Median latent period of tumour incidence (days) | Incidence of Metastases | |
|---|---|---|---|---|---|---|---|---|---|
| | | | Metastasis +ve | Metastasis −ve | *n* | % | | *n* | % |
| Rama 37 | – | 25 | – | 1.93 ± 0.37 | 18 | 72 | 15 (12–16) | 0 | 0 |
| RMP1 | G-cell | 23 | 2.15 ± 0.13 | 2.01 ± 0.39 | 22 | 95.6 | 14 (10–19) | 2 | 8.7 |
| RMP2 | AT6.1 cell | 20 | 2.07 ± 0.34 | 2.21 ± 0.41 | 20 | 100 | 13 (9–19) | 5 | 25 |
| RMP3 | Rama 37 cell | 21 | – | 2.24 ± 0.49 | 19 | 90 | 14 (11–17) | 0 | 0 |

fragments responsible for the metastatic behaviour of rat prostate cancer cells may be identified. Furthermore, this tested assay system is now available to be applied to identify metastasis-promoting sequences contained within genomic DNA from any human malignancy.

### MATERIALS AND METHODS

### Preparation of tagged donor genomic DNA

Parental Rama 37 mammary epithelial cells and donor Dunning rat prostatic carcinoma G and AT6.1 cells were each grown to confluence in 15-cm-diameter Petri dishes, washed with phosphate-buffered saline (PBS) at room temperature and harvested by scraping with a sterile, siliconized rubber policeman into 30-ml sterile, siliconized 30-ml Corex tubes. High molecular weight genomic DNA was isolated using a DNA isolation kit (Flowgen, UK). Partial digestion of the high molecular DNA with *Hin*dIII was performed to avoid significant damage to genes of potential interest (Sambrook et al, 1989*a*). Before large-scale digestions were undertaken, a series of pilot reactions were performed to digest 1 μg of genomic DNA using different amounts of *Hin*dIII to determine the most suitable concentration of the enzyme to achieve fragments in the size range 5–25 kb (Figure 1).

A short synthetic double-stranded DNA sequence tag was used to flank *Hin*dIII-digested fragments of genomic DNA (Chen et al, 1997). Two complementary oligonucleotides (upper and lower strands; Figure 2) were chemically synthesized and annealed by heating at 65°C for 2 min followed by slowly cooling to room temperature. The unique 22-base sequence engineered within the synthetic oligonucleotides acts as both primers for subsequent PCR reactions. Its structure prevents both mispriming and oligomerization when used as a PCR primer to amplify the tagged fragments of genomic DNA.

The *Hind*III partially digested genomic DNA fragments from the G cells, from the AT6.1 cells and from the Rama 37 cells were ligated to the synthetic oligonucleotide tags at a molecular ratio of 1:20 using bacterial T4 ligase. The reactions were performed at 16°C overnight. Excess unlinked oligonucleotides, and the 'flanked' DNA fragments were separated by agarose gel electrophoresis in low melting point agarose. Genomic DNA fragments linked to their synthetic flanking sequence were recovered with a GENECLEAN kit (Bio 101, USA) and stored in absolute ethanol at – 20°C until required for transfection studies.

**Figure 4** Phase-contrast morphology of cells in monolayer tissue culture. (**A**) Parental Rama 37 cells showing a distinct epithelial morphology similar to that identified in the primary inoculants. (**B**) RMP 1 cell-line derived from a pulmonary metastasis. (**C**) Cell line established from a pulmonary metastasis produced by RMP 2 cells. (**D**) Cell line established from a cardiac metastasis produced by RMP 2 cells. In contrast to the appearance of the original cells, those of all three metastatic lines are small, fusiform in appearance and are poorly cohesive

### Co-transfection of Rama 37 cells with pSV2neo and tagged DNA fragments

Calcium phosphate precipitation was used to perform the transfection experiments (Jamieson et al, 1990*a*; Davies et al, 1994). Exponentially growing parental Rama 37 cells were harvested by scraping and seeded at a density of 0.5–0.75 × $10^6$ cells per 10 ml of Dulbecco's modified Eagle medium (DMEM) in each 10-cm-diameter Petri dish. After incubation overnight at 37°C, spent medium was replaced with fresh DMEM at 4 h before transfection. Donor DNA fragments (18 μg) from each of the cell-lines G, AT6.1 or Rama 37 and pSV2*neo* plasmid DNA (2 μg) were precipitated with calcium phosphate by bubbling air through an 0.025 M solution of Hepes containing 0.14 M sodium chloride, 0.75 mM sodium hydrogen phosphate and 0.125 M calcium chloride (pH 7.1). The precipitated DNA calcium phosphate complex was

© Cancer Research Campaign 1998 

**Figure 5** Development of systemic metastases in syngeneic rats after inoculation of Rama 37 cells transfected with *Hin*dIII-digested genomic DNA into the mammary fat pad. (**A**) Lung metastasis produced by RMP 1 cells. (**B**) Lung metastasis produced by RMP 2 cells. (**C**) Lung and cardiac metastases produced by RMP 2 cells recovered in 1 ml of the solution and added directly to 10 ml of DMEM medium covering 1 × 10⁶ cells in each Petri dish. After incubation for 12 h at 37°C, the medium was replaced by fresh DMEM containing 10% (v/v) DMSO (Spandidos and Wilkie, 1984) at room temperature for 1.5 min. The medium was removed, the cells washed once with fresh medium, and then incubated at 37°C for 24 h. The cells were passaged at a 1:10 dilution in a selective medium comprising DMEM containing Geneticin-G418 at the concentration of 1 mg $ml^{-1}$. Thereafter, this medium was replaced every 3 or 4 days. Cell culture was continued for 1 week after colonies became visible. When the colonies had grown to more than 1.5–3.5 mm in diameter, the cells were harvested, pooled and cultured continuously in identical selection medium until large numbers of cells were obtained. The three cell pools obtained by co-transfection of parental Rama 37 cells with fragments of genomic DNA from prostatic G cells, AT6.1 cells or benign mammary Rama 37 cells were designated RMP1, RMP2 and RMP3 respectively (Figure 3).

### Tumorigenicity and metastatic ability of transfected cells

The control Rama 37 cells and the three transfectant cell lines were harvested by treatment with EDTA/trypsin and washed once with PBS. Four groups, each comprising 25 female Wistar–Furth (OLA strain) rats, 4- to 6-week-old, were used to assay the control parental Rama 37 cells and the three transfected cell lines for their tumorigenicity and metastasizing ability (Table 1). Each animal was inoculated subcutaneously into the right inguinal mammary fat pad with a depot of 0.2 × $10^6$ cells in PBS. Animals dying soon after the ulceration of their primary tumour, or after inadvertent inoculation of tumour cells into sites extending beyond the anatomical confines of the mammary fat pads, were excluded from the study. All surviving animals retained within the study were autopsied at 3 months after inoculation. The lungs, liver, spleen, kidney, heart and axillary lymph nodes in each animal were examined for gross metastases. Small pieces of tissue were taken from organs containing macroscopically visible metastases. If an organ was suspected of containing metastases, it was minced and used to re-establish tumour cells in culture in the presence of G418 to confirm that they were of transfectant origin.

**Figure 6** Histological appearance of primary tumours produced by transfected Rama 37 cells and the metastases that developed in rats inoculated with different transfectant cells. (**A**) Appearance of primary RMP 2 tumours produced by transfected Rama 37 cells containing rat prostatic cancer genomic DNA. The tumour is composed of small, predominantly spindle-like tumour cells, with no glandular pattern. (**B**) Low-power appearance of 'cannonball' metastasis in the lung produced by RMP 1 cells. (**C**) RMP 1 tumour cells entering the vascular compartment from within one of the primary deposits. (**D**) Histological appearance of RMP 2 cells invading cardiac muscle. The tumour is composed of highly undifferentiated epithelial cells that show no organizational features

### Histological examination

Samples of primary tumours, and all tissues taken at autopsy, were fixed in Methacarn (methanol–inhibisol–acetic acid; 6:3:1), or in

 © Cancer Research Campaign 1998

**Table 2** Incidence of metastases produced by metastasis-derived cell lines

| Cell line | Number of animals | Latent period | Number of animals with metastases | Animals with metastases (%) |
|---|---|---|---|---|
| RMP1a-Lu | 9 | 14 | 1 | 11 |
| RMP1b-Lu | 7 | 13 | 1 | 14.3 |
| RMP2c-Lu | 8 | 10 | 2 | 25 |
| RMP2c-H | 8 | 10 | 3 | 37.5 |
| RMP2d-Lu | 4 | 10 | 1 | 25 |

RMP1a-Lu, lung metastasis produced in animal 'a' by RMP1 cells (derived from Dunning G Cells); RMP1b-Lu, lung metastasis produced in animal 'b' by RMP1 cells (derived from Dunning G cells); RMP2c-Lu, lung metastasis produced in animal 'c' by RMP2 cells (derived from Dunning AT6.1 cells); RMP2c-H, cardiac metastasis produced in animal 'c' by RMP2 cells (derived from Dunning AT6.1 cells); RMP2d-Lu, lung metastasis produced in animal 'd' by RMP2 cells (derived from Dunning AT6.1 cells).

neutral buffered formol saline, processed conventionally, embedded in paraffin wax, sectioned onto glass slides and stained with haematoxylin and eosin (Dunnington et al, 1983). Sections of each tissue were examined by two independent observers.

### Tissue culture

Rat mammary epithelial cell line Rama 37, and all derived cell lines (Figure 4), were routinely cultured in DMEM, containing 5% (v/v) fetal calf serum, 50 ng $ml^{-1}$ hydrocortisone and 50 ng $ml^{-1}$ insulin (Dunnington et al, 1984). The reversible toxic effects of Geneticin-G418 (Gibco Bio-Cult, Paisley, UK) on Rama 37 cells (Southern and Berg, 1982) was optimal at 0.8–1.0 mg $ml^{-1}$. The primary culture technique used to re-establish tumour cells in vitro was similar to that described by Dunnington et al, 1984. A small piece of non-necrotic tissue containing metastatic nodules or micrometastases was minced using sterile scalpel blades. The tissue was homogenized in 10 ml of DMEM with a Teflon pestle and the suspension allowed to sediment by standing at room temperature for 1 minute. The supernatant was removed and incubated at 37°C. The sediment was incubated in a rotary mixer, for 1 h at 37°C, in 20 ml of DMEM containing 2 mg $ml^{-1}$ collagenase (type I). The digest was allowed to sediment for 1 min before the supernatant from this step was combined with that from the homogenization step. Both were then centrifuged at 800 rpm for 5 min at room temperature in an MSE bench-top centrifuge. The cell pellet was washed in 20 ml of DMEM, resuspended in 10 ml of DMEM containing Geneticin-G418 at a concentration of 1 mg $ml^{-1}$, and plated in 5-cm-diameter Petri dishes.

### Nucleic acid hybridization

Southern blot hybridization was performed according to Sambrook et al (1989*b*). Total genomic DNA extracted from cells was partially digested with *Hin*dIII to generate a range of fragments of molecular weight from 5 kb to 25 kb. Aliquots of 10 μg from each preparation were subjected to agarose gel electrophoresis to separate the DNA fragments. Standard DNA fragments were used as molecular weight markers. The fragments of DNA separated by electrophoresis were excised from the gel and depurinated in 0.2 M hydrochloric acid for 10 min, denaturated in 0.4 M sodium hydroxide and 1 M sodium chloride for 45 min and neutralized in 0.5 M Tris-HCl containing 1 M sodium chloride (pH 7.2) for 15 min. DNA fragments were transferred onto a nylon membrane (Amersham International, Amersham, UK), according to the manufacturer's protocol. Cross-linking of DNA to the membrane was achieved by exposing the blot to a 302 nm UV transilluminator for 3 min.

An 881-bp fragment of *neo* DNA was obtained by digesting the pSV2*neo* vector with *Hin*dIII and *Bss*HII. This cDNA probe was labelled with [$\alpha^{32}$P]dCTP to a specific activity of between $6 \times 10^8$ and $1 \times 10^9$ d.p.m. per μg DNA using random primed synthesis (Feinberg and Vogelstein, 1983). The oligonucleotide probe (the lower strand of the short DNA synthetic linker) was labelled at the 5′ terminus with [$\alpha^{32}$P]ATP, to a specific activity of $2.4 \times 10^7$ d.p.m. per μg, using a polynucleotide kinase (Pharmacia, Milton Keynes, UK). For detection of the *neo* gene and the presence of the short DNA linker, the radioactively labelled probes were incubated with the DNA blots in a hybridization oven (Techne HB-10, Philip Harris, UK) for at least 16 h, at the predetermined optimum conditions. The hybridized probes were detected by autoradiography at – 70°C using Kodak X-AR5 or X-O-Mat S film.

### RESULTS

### Preparation of tagged donor genomic DNA

Approximately 500 μg of high molecular weight genomic DNA was extracted from each of the weakly and highly metastatic rat prostate cell lines, the G cells and AT-6.1 cells, respectively, and the benign Rama 37 cells. To prevent damage to gene sequences of possible interest, a partial enzymatic digestion of DNA was performed by *Hin*dIII cleavage so that the size of the resulting fragments ranged between 5 and 25 kb (Figure 1). DNA fragments of an ideal size (5–25 kb) were obtained at a ratio of 0.2 unit enzyme per 1 μg of DNA, and digested at 37°C for 1 h. After recovery of the partially digested genomic DNA, these fragments were covalently 'tagged' with short synthetic DNA sequences (Figure 2) using bacterial T4 ligase. After the ligation reaction, excess unlinked 'tags' were separated from the genomic DNA by electrophoresis in low melting agarose gel. About half of the 'tagged' DNA fragments were recovered from the agarose gel.

### Co-transfection of Rama 37 cells with pSV2neo and tagged DNA fragments

When cultured in Geneticin-containing selection medium, all three co-transfectants yielded small colonies that became visible 10 days after co-transfection of Rama 37 cells with pSV2*neo* plasmid DNA together with, but unlinked, donor DNA fragments from Dunning G cells, AT6.1 cells and a control of DNA from Rama 37 cells (Figure 4). Transfection frequencies of DNA from the three

© Cancer Research Campaign 1998

**Figure 7** Southern blot detection of donor DNA fragments in the malignant variants and in their parental transfectant cells, using the lower strand of the synthetic oligonucleotide 'tag' as a probe. (**A**) Transfectant pooled cells: (1) DNA from the control Rama 37 cells; (2) DNA from RMP3 cells; (3) DNA from RMP 1 cells; (4) DNA from RMP 2 cells. (**B**) Metastatic variants: (1) DNA from RMP 1a-Lu; (2) DNA from RMP 2a-Lu; (3) DNA from RMP 2b-Lu; (4) DNA from RMP 2c-Lu; (5) DNA from RMP 2-H; (6) DNA from the control Rama 37 cells

**Figure 8** Southern blot analysis to confirm the presence of the drug resistance *neo* gene in the transfectant cell pools and in the malignant cell lines established from the metastases, using an 881 bp *neo* cDNA fragment excised by Hind III and BSSH II to hybridize the genomic DNA from different cells. Lane 1, RMP 1a-Lu; lane 2, RMP 1b-Lu; lane 3, RMP 2a-Lu; lane 4, RMP2b-Lu; lane 5, RMP 2-H; lane 6, RMP 1; lane 7, RMP 2; lane 8, RMP 3, Lane 9, Rama 37 (control)

donor cell lines were $1.44 \times 10^{-5}$, $2.16 \times 10^{-5}$ and $1.98 \times 10^{-5}$ respectively. There were no significant differences between the growth rates of the transfected cells and parental Rama 37 cells (data not shown).

### Tumorigenicity and metastatic ability of transfected cells

A schematic diagram is provided (Figure 3) to illustrate the relationship of the parental Rama 37 cells with the transfectant cell lines, and their resultant metastases. The mean latent period for in vivo growth of primary tumours by non-transfected parental Rama 37 cells and three groups of transfectants was not significantly different from one another ($P < 0.5$), ranging in their means from 13 to 15 days (Table 1). At autopsy, the primary tumours in each group varied from 1.5 cm to 3.5 cm in diameter. Of the animals inoculated with non-transfected parental Rama 37 cells, 72% developed palpable primary tumours by 3 months after inoculation. In the three transfectant groups, the numbers of tumours were significantly greater than in those not transfected with Rama 37 cells, ranging from 90% to 100% (Fisher's exact test, $P < 0.05$).

No metastases were identified either macroscopically or histologically in the two control groups of animals inoculated with parental (non-transfected) Rama 37 cells or with Rama 37 cells autotransfected with Rama 37 genomic DNA (RMP3). In contrast, metastases occurred in both transfectant cell lines containing genomic DNA from either low- or high-metastatic prostatic carcinomas. Pulmonary mestastases developed in 2 out of 22 tumour-bearing animals in group RMP1 (G-cell donor DNA transfectants). Of these, one metastatic deposit was identified macroscopically at autopsy (Figure 5A). The second deposit was discovered on histological examination. In group RMP2 (AT6.1-cell donor DNA transfectants) 5 out of 20 tumour-bearing animals developed pulmonary metastases (Figure 5B). Strikingly, a cardiac metastasis localized in the apex of the heart (Figure 5C) occurred in one of the five animals in which lung metastases also developed.

The average sizes of primary tumours among all four groups of animals at the end of the experiments were not significantly different from one another ($P < 0.03$). The average size of the primary tumours in animals inoculated with Rama 37 cells was $1.93 \pm 0.37$ cm diameter, compared with $2.01 \pm 0.39$ cm, $2.21 \pm 0.41$ cm and $2.24 \pm 0.49$ cm for those developed in rats inoculated with RMP1 (G-cell DNA transfectants), RMP2 (AT6.1 cell DNA transfectants) and RMP3 (benign cell DNA transfectants) respectively. The average sizes of the primary tumours that produced metastases were $2.15 \pm 0.13$ cm and $2.07 \pm 0.34$ cm in rats inoculated with RMP1 and RMP2 cells, respectively, and compared with the average sizes of $2.01 \pm 0.39$ cm and $2.21 \pm 0.41$ cm for primary tumours which did not produce metastases, but which were developed from the identical transfectant cell stock (RMP1 and RMP2) respectively. The sizes of the secondary tumours varied greatly. The cardiac metastasis produced by RMP2 cells (AT6.1-cell DNA transfectants) was 6 mm in diameter. The sizes of numerous lung metastases developed in the four animals inoculated with RMP2 cells varied from 1 mm to 3 mm in diameter. Both lung metastases produced by RMP1 cells (G-cell DNA transfectants) in the two affected rats were not visible and were much smaller than those produced by RMP2 cells. One of the RMP1 metastases was found by microscopic examination of histological sections, the other was identified by primary tissue culture.

Apart from a few animals which developed ulcerated tumours, and hence were excluded from further experiments, the rats bearing only primary tumours (no metastases) did not exhibit visible symptoms of illness during the period of the experiments. For the four animals bearing metastases after inoculation of RMP2 cells (AT6.1-cell DNA transfectants), symptoms of illness

© Cancer Research Campaign 1998

appeared gradually and increased in severity during the late stage of the experiments. This was particularly apparent in the rat bearing the cardiac metastasis. Before the end of the experiment, this animal exhibited slow movements, increased cardiac rate, dyspnoea, and failed to react to external stimulation. For the two animals bearing lung metastases developing from the RMP1 cells (G-cell DNA transfectants), no systemic symptoms were observed.

### Histological examination

The primary tumours that developed in the two control groups (parental Rama 37 and RMP3) appeared morphologically indistinguishable from one another. Both groups of tumours were encapsulated and comprised medium-sized and cytologically benign epithelial cells arranged into partially solid and partially gland-like patterns. In contrast, the primary tumours that developed in the two groups of animals transfected with prostatic carcinoma DNA (RMP1 and RMP2) were composed of small and highly malignant looking spindle-shaped tumour cells (Figure 6A) that extensively invaded into adjacent stroma and muscle.

The metastatic pulmonary lesions from the prostatic carcinoma DNA transfectants ranged in size from 0.5 mm to 3 mm. The pulmonary metastases produced by RMP1 (G-cell donor DNA transfectants) consisted of nodules scattered throughout the lung parenchyma (Figure 6B). Their likely origin from the primary deposits was visible in several of the animals (Figure 6C). In contrast, most of the nodules produced by RMP2 (AT6.1-cell donor DNA transfectants) were either localized on the subpleural surfaces of the lungs or located within the lung parenchyma adjacent to pulmonary vessels. Additional metastases from these tumours were present within mesenteric lymph nodes. These deposits were less numerous but larger (3–5 mm diameter) than those produced by RMP1 (1.5–3 mm diameter).

The metastatic nodules produced by both RMP1 and RMP2 occurred as non-encapsulated tumours composed of small fusiform malignant cells together with an infiltration of large mononuclear and giant multinucleate cells of malignant cytology. The cardiac metastasis produced by RMP2c-H comprised a non-encapsulated mass of fusiform tumour cells together with some tumour giant cells that infiltrated between cardiac myocytes and effaced all normal tissues (Figure 6D).

### Development of cell lines from metastases and their metastatic potential

One pulmonary metastasis produced by RMP1, three pulmonary metastases produced by RMP2 and the cardiac metastasis produced by RMP2, were recovered at autopsy and successfully re-established in primary tissue culture. Three additional metastases discovered on histological examination initially grew in tissue culture, but were too small to be rescued. The five successfully established metastatic variants were designated as RMP1a-Lu (lung metastasis 'a' produced by RMP1), RMP2a-Lu, RMP2b-Lu, RMP2c-Lu (lung metastases 'a', 'b' and 'c' produced by RMP2) and RMP2c-H (heart metastasis 'c' produced by RMP2) (Figure 4).

In tissue culture, the cellular morphology of all the metastatic variants appeared similar to one another, although very different from that of the parental Rama 37 cells. Whereas the original Rama 37 epithelial cells were large and predominantly cuboidal, with only a very few elongated cells arising at the peripheries of the colonies, the transfectant tumour cells from the metastases typically appeared as small and loosely adherent spindle cells. When these five new cell-lines were reinoculated individually into five groups of syngeneic rats (second round transformants), 100% tumorigenicity was observed (Table 2). All five groups of animals developed metastases; further confirming the metastatic nature of these Rama 37 variants.

### Detection of the donor DNA fragments and drug resistance gene

Genomic DNA extracted from RMP1, RMP2 and RMP3 cells, digested completely with *Hin*dIII and subjected to Southern blot hybridization using the lower strand of the short DNA 'tag' as the probe to detect donor DNA fragments, confirmed the presence of a few bands in all three cell pools (Figure 7A). In all five metastatic cell lines, the presence of the short synthetic DNA linker was confirmed. No band was detected in the genomic DNA from control (untransfected) Rama 37 cells. The patterns of additional bands in the three transfectant cell pools (RMP1–RMP3) were not identical, the intensities of different bands varying between the three cell lines. Within the RMP1 cell lines, only two bands with sizes of approximately 10 kb and 3 kb were identified. RMP2 cell lines revealed three bands with sizes of approximately 4.5 kb, 3 kb and 1.3 kb. The RMP3 cell line contained a smear of bands in the range from 5 kb to 2.5 kb with a distinct band at 1.3 kb. One of the bands detected in all five cell lines established from metastases (Figure 7B) was constant at about 1.3 kb. When an 811-bp-long *neo* gene fragment excised from pSV2*neo* plasmid by *Hin*dIII and *Bam*HII was used as a probe to hybridize similar blots, a series of bands was detected in the genomic DNA from all three pooled cell transfectants and from all five new metastatic cell lines (Figure 8). No band was detected in the negative control DNA from untreated Rama 37 cells.

Because both ends of the transfected DNA fragment are 'tagged' by the same short DNA molecule, only one primer is needed for performing PCR to amplify the 'tagged' DNA fragments (see Figure 2). Using either the up or the low strand of the short DNA 'tag' as a primer to carry out the PCR experiment on the total genomic DNA extracted from the cell lines established from the metastases, we have amplified and isolated 13 DNA fragments from within the genome of these metastatic cell lines. We are currently conducting further studies on the capabilities of these 13 DNA fragments in promoting metastasis in Rama 37 cells, either in a collective or an individual manner.

### DISCUSSION

The combined techniques of DNA transfection and cell selection using a drug resistance plasmid together provide a powerful and important strategy in the study of mechanisms of tumorigenesis and metastasis. Using this general approach, transformation of mouse NIH 3T3 fibroblasts previously led to identification of activated cellular oncogenes such as the *ras* family. However, oncogenes that transform NIH 3T3 fibroblasts and produce fibrosarcomas (Land et al, 1983) do not produce metastases when transfected into rat benign epithelial cells (Davies et al, 1993). Earlier work on the generation of metastatic variants of Rama 37 cells by transfection of genomic DNA from rat and human breast carcinoma cells confirmed the validity of DNA transfection as a technique with which to assay the metastatic capability of genomic

© Cancer Research Campaign 1998

DNA fragments (Jamieson et al, 1990*a*; Davies et al, 1994), as well as that of a variety of cellular oncogenes (Bernstein and Weinberg, 1988), and the gene for calcium-binding protein p9Ka (Jamieson et al, 1990*b*; Davies et al, 1993). Using this technique, several DNA fragments closely associated with metastasis in human breast cancer cell lines have been identified (Chen et al, 1997). In this system, the transfected DNA responsible for inducing metastasis does not code for growth-promoting oncogenes such as *ras* etc. as these consistently fail to induce metastasis, in contrast to genes such as that for p9Ka (Jamieson et al, 1990*a*; Davies et al, 1993). Although the Rama 37 cell system has been successfully used to assay genomic DNA from breast cell lines, it has not been used previously to examine the metastatic ability of DNA from other sources.

In this current work, we describe the first use of this system to transfect prostatic carcinoma genomic DNA and to generate new metastatic variants of Rama 37 cells. Dunning rat G cells are only very weakly metastatic. When this cell line was inoculated into immunocompetent Copenhagen rats, less than 5% of animals developed metastases. However, when highly metastatic AT6.1 cells were inoculated into the same host strain, more than 75% of rats developed metastases (Isaacs et al, 1986). In the present work, transfection of total genomic DNA from weakly metastatic G cells into benign Rama 37 cells induced metastases in 8.7% of the experimental animals, whereas the DNA from the highly metastatic AT6.1 cells induced metastases in 25% of the experimental animals. The mean size of the secondary metastatic tumours produced by AT6.1-cell DNA transfectants (RMP2) was larger than the mean size of those induced by G-cell DNA transfectants (RMP1). All rats bearing metastatic tumours produced by AT6.1-cell DNA transfectants (RMP2) exhibited symptoms of systemic illness that increased during the later stage of the experiments. However, the animals bearing metastases induced by G-cell DNA transfectants (RMP1) did not exhibit systemic illness. This observation indicates that the metastatic capability of the DNA of AT6.1 cells was greater than that of the G cells, which correctly reflects the biological characteristics of the Dunning G cell line and of the AT6.1 cell line in our experimental system.

The average sizes of the primary tumours developed in each of the four groups of animals were very similar ($P < 0.03$). The average sizes of primary tumours that produced metastases were $2.15 \pm 0.13$ cm and $2.07 \pm 0.34$ cm in rats injected with RMP1 and RMP2 cells, respectively, when compared with the average sizes of $2.01 \pm 0.39$ cm and $2.21 \pm 0.41$ cm, respectively, of primary tumours that did not produce metastases but which were developed from the same stocks of transfectant cells (RMP1 and RMP2 respectively). The differences in average primary tumour sizes between the metastases-bearing animals and benign tumour-bearing animals was smaller than those between the animals within the same category. Therefore, in this system, the sizes of primary tumours did not relate to the incidence of metastasis. It is possible that the observation periods, and hence the numbers of obtained metastases, were arbitrarily limited by the growth of the primary tumours that remained in situ. However, in previous work during the development of this system, this observation period was increased to at least 12 months with no alteration in metastatic frequencies (Jamieson et al, 1990*a*). There is also the possibility of bias in relying on one single animal system for the detection of genes and/or fragments of DNA that can cause metastatsis. However, previous studies using the equivalent all mammary system have identified two genes, p9Ka (S100A4) (Davies et al, 1993) and osteopontin (Oates et al, 1996), that have been shown subsequently to induce metastasis in other rodent systems (Ambartsumian et al, 1996; Davies et al, 1996) or in which reduction of their expression using antisense technology blocks the development of the metastatic state in other rodent systems (Behrend et al, 1994; Gardner et al, 1994; Grigorian et al, 1993).

The incidence of tumours produced in the three groups of animals inoculated with transfected cells was not significantly different. However, this incidence was significantly higher than that produced by the non-transfected, parental, Rama 37 cells. Although the tumorigenicity of RMP3 cells (Rama 37 cells transfected with Rama 37 DNA) was higher than that of parental (non-transfected) Rama 37 cells, the transfectants did not produce metastases in vivo. In contrast, RMP1 (Rama 37 cells transfected with low metastatic prostatic G-cell DNA) produced lung metastases in two animals; RMP2 (Rama 37 cells transfected with high-metastatic prostatic AT6.1-cell DNA) produced lung metastases in five animals. Within this latter group, one animal also developed a cardiac metastasis. This observation further confirms that production of metastases by RMP1 and RMP2 cells is dependent upon the particular source of the transfected DNA, rather than on the transfection process itself.

One potential problem encountered in DNA transfection experiments has been to determine the most appropriate size for the donor DNA fragments. Sequences that are too large reduce transfection efficiency and increase the likely difficulty of their rescue from recipient cells. Fragments that are too small risk damage to potential target gene(s). In early transfection studies with 3T3 cells that led to identification of *ras* genes, a few restriction enzymes were used to fragment the donor DNA to avoid cutting within the target region of the DNA (Cooper et al, 1980). In the current study, *Hin*dIII was chosen as the single enzyme to cleave donor DNA. Partial digestion of genomic DNA with *Hin*dIII (at 0.2 unit $\mu g^{-1}$) yielded a smear of bands with sizes ranging from 200 to 25 kb, although the majority of bands were located in the region between 5 kb and 25 kb (Figure 1). The observation that transfection of fragmented *Hin*dIII genomic DNA from G cells and AT6.1 cells results in metastatic transfectants confirms that potential DNA targets have not been completely damaged by *Hin*dIII partial digestion, as the metastatic capability of DNA in the size range was not destroyed.

Recovery of donor DNA fragments from within the genome of cells established from metastatic tumours is the major logistical difficulty to be overcome in DNA transfection experiments. In earlier studies, human DNA transfected into 3T3 cells has been recovered by identification of human specific *Alu* sequences (Gate et al, 1995; Hayle et al, 1993). Although several oncogenes have been identified by this method, the risk involved in this strategy is that, although *Alu* sequences are scattered throughout the human genome they are not contained in every fragment of human DNA (only 50% according to probability and depending on the sizes of the fragments). Conversely, the current work has used the specific strategy of 'flanking' all fragments of donor DNA with short synthetic DNA oligonucleotide sequences before transfection (Chen et al, 1997). The short synthetic DNA sequences were engineered in such a manner that not only could they be ligated to the fragments of genomic DNA released by digestion with *Hin*dIII but also were able to ligate both ends of the fragments when cut back by *Hin*dIII. This approach provides a powerful and highly specific strategy for recovering particular donor DNA fragments from the

 © Cancer Research Campaign 1998

genome of cells derived from metastases. Screening a DNA library constructed from the genomic DNA of these new malignant cells will enable us to identify those 'tagged' fragments, using either the upper or lower strand of the synthetic flanking DNA sequences as a probe. Furthermore, the sequence comprising 22 of the 45 bases within the upper strand of the synthetic oligonucleotide flanking sequence has been specifically engineered for use as a PCR primer in subsequent amplification procedures (Figure 2). The inherent symmetry of the designed sequences is such that only this single primer sequence is required to amplify target genomic DNA fragments flanked by these particular synthetic oligonucleotides. Therefore, PCR-based sequencing techniques may now be applied directly to genomic DNA extracted from the newly established metastatic cells and will provide a direct approach to isolating those DNA fragments responsible for metastasis in the rat model system used here. Furthermore, this tested assay system is now available to be applied to identify metastasis-promoting sequences contained within genomic DNA from any human malignancy.

Initially, we were concerned that too many fragments of genomic DNA might be recovered and that these would require further selection to identify the most important at generating the metastatic phenotype. However, preliminary experiments using PCR have identified only 13 distinct fragments in all five metastatic variants established from both lung and cardiac metastases. There are two likely possibilities for this relatively low number: first, only those individual cells harbouring the target fragments can metastasize to secondary sites. Growth of these cells in vivo has provided a powerful mechanism by which individual cells with metastatic capability are selected, and has filtered-out the majority of cells containing non-specific DNA fragments. Southern blot data show a significant reduction in the number of donor DNA fragments in the five new metastatic cell-lines when compared with the initial three pools of transfectant cells (Figure 7A). Second, the number of fragments that may be successfully accumulated by a single cell, while maintaining general stability of its genome, is limited. The cells comprising an individual metastatic tumour may have originated from a single cell within the primary tumor cell pool. Thus, mechanisms to ensure genomic stability and survival of transfected cells, together with their growth in syngeneic animals, provides a dual process of selection by which only those cells containing the transfected genomic DNA that is most important for metastatic dissemination of the cells to become secondary tumours can survive. The structure of these 13 fragments recovered by direct PCR is currently being analysed. A further round of animal assays using these selected fragments will confirm which, either singly or in combination, is responsible for generating metastases in this model system.

Metastatic disease is the major cause of treatment failure and death from prostatic cancer. A series of genetic events is probably necessary before a prostatic tumour cell develops the capacity to metastasize (Sandberg, 1997). Such genetic changes include abnormal expression of metastasis-promoting genes in addition to a decrease in activities of tumour-suppressor genes. Chromosome-transfer studies have confirmed human chromosome 17q12–22 to contain a novel tumour-suppressor gene in this region (Murakami et al, 1995). Hybridization of the non-metastatic Dunning AT2.1 cell line with highly metastatic prostate carcinoma AT3.1 cells (Ichikawa et al, 1991) led to the discovery of a small metastasis-suppressor gene and its human counterpart, KAI1 located on human chromosome 11p11.2 (Dong et al, 1995). Conversely, after differential display analysis of cell lines with distinct behavioural phenotypes from within the Dunning rat prostatic carcinoma model, the protein thymosin β15 has been identified to be selectively elevated in the metastatic carcinoma cells (Bao et al, 1996) and its gene regarded as a possible 'metastasis gene'. A third putative metastasis-associated gene located on human chromosome 1 has been provisionally identified after linkage analysis (Smith et al, 1996). However, as yet, there are no experimental data to locate, or even to support the existence, of a particular gene at this site. In our own recent work, we found the expression of a calcium-binding protein p9Ka increases as the increasing metastatic characteristics of prostate epithelial cells (Ke et al, 1997). In this report, we now provide strong evidence for at least one metastasis-promoting locus in this rate model system of prostate cancer. We consider that elevated activity of this DNA, possibly in conjunction with other DNAs, might play an important role in promoting metastasis in prostate cancer. Such regions of DNA that may be ultimately responsible for positively stimulating cell migration and metastasis will be prime targets for biologically appropriate molecular therapeutic technique.

### ABBREVIATIONS

PCR, polymerase chain reaction; PBS, sodium phosphate-buffered saline; DMEM, Dulbecco's modified Eagle's medium; bp, base pairs; DMSO, dimethylsulphoxide.

### ACKNOWLEDGEMENTS

Professor CS Foster and Dr Y Ke acknowledge the generous support of the Prostate Cancer Cure Foundation and the North West Cancer Research Fund for providing the funding that has enabled this work to become established. We also thank Mr Alan Williams for his photographic assistance and Miss Jill Shaw for typing and editing the manuscript.

### REFERENCES

Ambartsumian NS, Grigorian MS, Larsen IF, Karlstrom O, Sidenius N, Rygaard J, Georgiev G and Lukanidin E (1996) Metastasis of mammary carcinomas in GRS/A hybrid mice transgenic for the mtsl gene. *Oncogene* **13**: 1621–1630

Bao L, Loda M, Janmey PA, Stewart R, Anand-Apte B and Zetter BR (1996) Thymosin beta 15: A novel regulator of tumor cell motility upregulated in metastatic prostate cancer. *Natl Med* **2**: 1322–1328

Behrend EI, Craig AM, Wilson SM, Denhardt DT and Chambers AF (1994) Reduced malignancy of ras-transformed NIH 3T3 cells expressing antisense osteopontin RNA. *Cancer Res* **54**: 832–837

Bernstein CS and Weinberg RA (1988) Correction to expression of the metastatic phenotype in cells transfected with human metastatic tumor DNA. *Proc Natl Acad Sci USA* **85**: 5581–5886

Boring CC, Squires TS, Tong T and Montgomery S (1994) Cancer Statistics, 1994. *CA-A Cancer J Clin* **44**: 7–26

Chen H, Ke Y, Oates AO, Barraclough R and Rudland PS (1997) Isolation of and the effector for metastasis-inducing DNAs from a human metastatic carcinoma cell line. *Oncogene* **14**: 1581–1588

Cooper GM, Okenquist S and Silverman L (1980) Transforming activity of DNA of chemically transformed and normal cells. *Nature* **284**: 181–188

Davies BR, Davies MPA, Gibbs FEM, Barraclough R and Rudland PS (1993) Induction of the metastatic phenotype by transfection of a benign rat mammary epithelial cell-line with the gene for p9Ka, a rat calcium-binding protein, but not with the oncogene EJ-*ras*-1. *Oncogene* **8**: 999–1008

Davies BR, Barraclough R and Rudland PS (1994) Induction of metastatic ability in a stably diploid benign rat mammary epithelial cell line by transfection with DNA from human malignant breast carcinoma cell lines. *Cancer Res* **54**: 2785–2793

© Cancer Research Campaign 1998

Davies MPA, Rudland PS, Robertson L, Parry EW, Jolicoeur P and Barraclough R (1996) Expression of the calcium-binding protein S100A4(p9Ka) in MMTV-neu transgenic mice induces metastasis of mammary tumours. *Oncogene* **13**: 1621–1637

Dong JT, Lamb PW, Rinker-Schaeffer CW, Vukanovic J, Ichikawa T, Isaacs JT and Barrett JC (1995) KAI 1, a metastasis suppressor gene for prostate cancer on human chromosome 11p11.2. *Science* **286**: 884–886

Dunnington DJ, Hughes CM, Monaghan P and Rudl and PS (1983) Phenotypic instability of rat mammary tumor epithelial cells. *J Natl Cancer Inst* **71**: 1227–1240

Dunnington DJ, Kim U, Hughes CM, Monaghan P, Ormerod J and Rudland PS (1984) Loss of myoepithelial cell characteristics in metastasizing rat mammary tumors relative to their non-metastasizing counterparts. *J Natl Cancer Inst* **72**: 455–466

Feinberg AP and Vogelstein B (1983) A technique for radiolabelling DNA restriction endonuclease fragments to high specific activity. *Anal Biochem* **132**: 6–13

Foster CS (1990) Predictive factors in prostatic hyperplasia and neoplasia. *Hum Pathol* **21**: 575–577

Foster CS and Abel PD (1992) Clinical and molecular techniques for diagnosis and monitoring of prostatic cancer. *Hum Pathol* **23**: 395–401

Gardner HAR, Berse B and Senger D (1994) Specific reduction on osteopontin synthesis by antisense RNA inhibits the tumorigenicity of transformed rat 1 fibroblasts. *Oncogene* **9**: 2321–2326

Gate CC, Belloni DR and Marin-Padilla M (1995) Acquisition and enhanced expression of the metastatic phenotype following transfections of genomic mouse tumor DNA containing human SCLC gene sequences. *Clin Exp Met* **13**: 203–217

Glenn J, McDonald D, Horetsky RL and Sexton FM (1988) Metastatic phenotype in murine cells transfected with human DNA. *J Surg Res* **44**: 382–390

Grigorian MS, Tulchinsky EM, Zain S, Ebralidze AK, Kramerov DA, Kriajevska MV, Georgiev G and Lukanidin EM (1993) The mts1 gene and control of tumor metastasis. *Gene* **125**: 229–238

Hayle AJ, Darling DL, Taylor AR and Train D (1993) Transfection of metastatic capability with total genomic DNA from human and mouse metastatic tumor cell-lines. *Differentiation* **54**: 177–189

Ichikawa T, Ichikawa Y and Isaacs JT (1991) Genetic factors and suppression of metastatic ability of prostatic cancer. *Cancer Res* **51**: 3788–3792

Isaacs JT, Isaacs WB, Feitz WFJ and Scheres J (1986) Establishment and characterization of seven Dunning rat prostatic cancer cell-lines and their use in developing methods for predicting metastatic abilities of prostatic cancer. *Prostate* **9**: 261–281

Jamieson S, Barraclough R and Rudland PS (1990*a*) Generation of metastatic variants by transfection of a non-metastatic rat mammary epithelial cell-line with DNA from metastatic rat mammary cell-lines. *Pathobiology* **58**: 329–342

Jamieson S, Barraclough R and Rudland PS (1990*b*) Transfection of nonmetastatic diploid rat mammary epithelial cell line with the oncogenes for EJ-ras-1 and Polyoma Large T Antigen. *Int J Cancer* **46**: 1071–1080

Ke Y, Jing C, Barraclough RB, Smith PH, Davies MP and Foster CS (1997) The expression of the calcium-binding protein, p9Ka, elevates as the increasing metastatic characteristics in rat prostate carcinoma cell lines. *Int J Cancer* **71**: 832–837

Krontiris TG and Cooper GM (1981) Transforming activity of human-tumor DNAs. *Proc Natl Acad Sci USA* **78**: 1181–1184

Land H, Parada LF and Weinberg RA (1983) Tumorigenic conversion of primary embryo fibroblasts requires at least 2 cooperating oncogenes. *Nature* **304**: 596–602

Murakami YS, Brothman AR, Leach RJ and White RL (1995) Suppression of malignant phenotype in a human prostate cancer cell-line by fragments of normal chromosomal region 17q. *Cancer Res* **55**: 3389–3394

Oates AJ, Barraclough R and Rudland PS (1996) The identification of osteopontin as a metastasis-related gene product in a rodent mammary tumour model. *Oncogene* **12**: 97–104

Radler-Pohl A, Pohl J and Schirrmacher V (1988) Selective enhancement of metastatic capacity in mouse bladder carcinoma cells after transfection with DNA from liver metastases of human-colon carcinoma. *Int J Cancer* **41**: 840–846

Reitsma PH, Rothberg PG, Astrin SM, Trial J, Barshavit Z, Hall A, Teitelnauer SL and Kahn AJ (1983) Regulation of myc gene expression in HL-60 leukemia cells by a vitamin-D metabolite. *Nature* **306**: 492–494

Sambrook J, Fritsch EF and Maniatis T (1989*a*) *Molecular Cloning. A Laboratory Manual*, 2nd edn. pp. 924–930. Cold Spring Harbor Laboratory Press: New York

Sambrook J, Fritsch EF and Maniatis T (1989*b*) *Molecular Cloning. A Laboratory Manual*, 2nd edn. pp. 947–958. Cold Spring Harbor Laboratory Press: New York

Sandberg AA (1997) Detection of chromosomal abnormalities in human prostate cancers and their pathological significance. In *Pathology of the Prostate*. WB Saunders: London.

Shih C, Shilo B, Goldford MP, Dannenberg S and Weinberg RA (1979) Passage of phenotypes of chemically transformed cells via transfection of DNA and chromatin. *Proc Natl Acad Sci USA* **76**: 5714–5718

Shih C, Padhy LC, Murray M and Weinberg RA (1981) Transforming genes of carcinomas and neuroblastomas introduced into mouse fibroblasts. *Nature* **290**: 261–264

Smith JR, Freije D, Carpten JD, Gronberg H, Xu J, Isaacs SD, Brownstein MJ, Bova GS, Guo H, Bujnovszky P, Nusskern DR, Damber J-E, Bergh A, Emanuelsson M, Kallioniemi OP, Walker-Daniels J, Bailey-Wilson JE, Beaty TH, Meyers DA, Walsh PC, Collins FS, Trent JM and Isaacs WB (1996) Major susceptibility locus for prostate cancer on chromosome 1 suggested by a genome-wide search. *Science* **274**: 1371–1374

Southern PJ and Berg P (1982) Transfection of mammalian cells to antibiotic resistance with a bacterial gene under control of the SV40 early region promoter. *J Mole Appl Genet* **1**: 327–341

Spandidos DA and Wilkie NM (1984) Expression of exogenous DNA in mammalian cells. In *Transcription and Translation: A Practical Approach*, Hames BD and Higgins SJ (eds), pp. 1–48. Oxford IRL Press: Oxford.

Van Roy FM, Messiaen L, Leibaut G, Goa J, Dragonetti CH, Fiers WC and Mareel MM (1986) Invasiveness and metastatic capability of rat fibroblast-like cells before and after transfection with immortalizing and transforming genes. *Cancer Res* **46**: 4787–4795

Vousden KH, Ecceles SA, Purvies H and Marshall CJ (1986) Enhanced spontaneous metastasis of mouse carcinoma cells transfected with an activated c-Ha-ras-1 gene. *Int J Cancer* **37**: 425–433

Waghorne C, Kerbel RS and Breitman ML (1987) Metastasis potential of SP1 mouse mammary adenocarcinoma cells is differentially induced by activated and normal forms of c-H-ras. *Oncogene* **1**: 149–155

Wallace JS, Fleming KA and Tarin D (1988) Tumorigenic transformation of mouse NIH 3T3 cells by transfection with plasmid pSV2neo. *J Pathol* **156**: 73–76

© Cancer Research Campaign 1998